# Peroxidase-like activity of magnetic poly(glycidyl methacrylate-*co-*ethylene dimethacrylate) particles

**DOI:** 10.1038/s41598-018-38012-5

**Published:** 2019-02-07

**Authors:** Beata A. Zasońska, Petr Šálek, Jitka Procházková, Sindy Müllerová, Jan Svoboda, Eduard Petrovský, Vladimír Proks, Daniel Horák, Ivo Šafařík

**Affiliations:** 1 Institute of Macromolecular Chemistry, Czech Academy of Sciences, Heyrovského nám. 2, 162 06 Prague 6, Czech Republic; 2 Department of Nanobiotechnology, Biology Centre, ISB, Czech Academy of Sciences, Na Sádkách 7, 370 05 České Budějovice, Czech Republic; 3 Institute of Geophysics, Czech Academy of Sciences, Boční II/1401, 141 00 Prague 4, Czech Republic; 4 Regional Centre of Advanced Technologies and Materials, Palacký University, Šlechtitelů 27, 783 71 Olomouc, Czech Republic

## Abstract

Poly(glycidyl methacrylate) (PGMA) is prone to modifications with different functional groups, magnetic fluids or direct coupling with biological molecules. The purpose of this research was to synthesize new magnetically responsive particles with peroxidase-like activity. Poly(glycidyl methacrylate-*co*-ethylene dimethacrylate) [P(GMA-EDMA)] particles containing carboxyl groups were obtained by emulsifier-free emulsion polymerization and hydrolysis and oxidation of PGMA with KMnO_4_, resulting in poly(carboxymethyl methacrylate-*co*-ethylene dimethacrylate) [P(CMMA-EDMA)] particles. Thionine (Th) was also attached to the particles [(P(CMMA-EDMA)-Th] via EDC/NHS chemistry to observe its effect on electron transfer during the oxidation reaction. Finally, the particles were coated with a nitric acid-stabilized ferrofluid in methanol. The resulting magnetic particles were characterized by several methods, including scanning and transmission electron microscopy, X-ray photoelectron spectroscopy, and vibrating sample magnetometry. The effect of EDMA on the P(CMMA-EDMA) particle size and size distribution was investigated; the particle size changed from 300 to 340 nm, and the particles were monodispersed with a saturation magnetization of 11 Am^2^/kg. Finally, the effects of temperature and pH on the peroxidase-like activity of the magnetic P(CMMA-EDMA) and P(CMMA-EDMA)-Th particles were investigated. The particles, which exhibited a high activity at pH 4–6 and at ∼37 °C, represent a highly sensitive sensor component potentially useful in enzyme-based immunoassays.

## Introduction

In recent decades, there has been increasing attention given to enzymes, including peroxidases, due to their great industrial potential. Peroxidases are of natural origin; they use H_2_O_2_ as an electron acceptor to catalyze oxidative reactions. However, their interesting catalytic activity can be compromised by various disadvantages, such as a tendency for denaturation, the limited recycling, laborious preparation, and high cost^1^. To overcome these drawbacks, peroxidase mimics were discovered, which were of great interest for use in many areas, such as soil detoxification and bioremediation of wastewater, as biosensors, and as diagnostic ELISA kits in analytical systems for the determination of glucose, lactose, glutamate, alcohols, cholesterol, choline, uric acid, pathogens, etc.^2–6^. Examples of peroxidase mimics that possess a good thermal stability, are easy to prepare, and provide tunable catalytic activity include chelates of manganese, CeO_2_-montmorillonite, magnetite-polymer nanocomposites, graphene oxide, carbon nanotubes/dots, mesoporous silica-gold nanoparticles and gold nanoclusters^7–12^. Neat iron oxide nanoparticles or their advanced composites with activities similar to those of natural peroxidases have been studied for the targeting and visualizing of tumor tissues, sensor development, isolation and purification of biological compounds, etc.^13^. The presence of Fe^2+^ ions in magnetite plays a crucial role in its peroxidase-like activity^11,14^. The peroxidase-like activity is not restricted only to magnetite but also to other Fe oxides, such as hematite (α-Fe_2_O_3_), maghemite (γ-Fe_2_O_3_), and complex biological structures containing iron oxide (e.g., magnetoferritin)^15–17^.

As mentioned above, the described composite artificial peroxidase mimetics include only polymer-coated oxides but not magnetic iron oxide-coated polymer particles. Among the different micro- and nanomaterials, special attention is paid to magnetic poly(glycidyl methacrylate) (PGMA) particles of various sizes that can be easily prepared by emulsion or dispersion polymerization in the presence of a ferrofluid^18^. PGMA has an advantage due to the availability of oxirane groups, which simplifies further functionalization and enables separations^19–21^, wastewater treatment^22^, quantification of markers of autoimmune diseases^23,24^, etc. However, the possible peroxidase-like activity of these magnetic polymer particles has been overlooked, even though magnetite-containing particles combined with horseradish peroxidase have found some interesting applications in immunoassays^25,26^.

In our previous study, ELISA immunosensor, including poly(carboxymethyl methacrylate) (PCMMA) particles, obtained by hydrolysis and oxidation of PGMA, was prepared by emulsifier-free emulsion polymerization. These PCMMA particles were modified with horseradish peroxidase enzyme and the electron mediator, thionine, resulting in an ELISA immunosensor for electrochemical detection of H_2_O_2_^27^. In this work, poly(glycidyl methacrylate-*co*-ethylene dimethacrylate) [P(GMA-EDMA)] particles were synthesized and the effect of EDMA concentration in the reaction feed on the particle size was investigated. Hydrolyzed P(GMA-EDMA) was oxidized to introduce carboxyl groups, yielding poly(carboxymethyl methacrylate-*co*-ethylene dimethacrylate) [P(CMMA-EDMA)] particles. This was followed by covalent attachment of thionine (Th), modification with ferrofluid, and the determination of peroxidase-like activity of magnetic P(CMMA-EDMA), magnetic P(CMMA-EDMA)-Th (denoted as Mag-P(CMMA-EDMA) and Mag-P(CMMA-EDMA)-Th,) particles.

## Materials and Methods

### Chemicals

Ethylene glycol dimethacrylate (EDMA), glycidyl methacrylate (GMA), *N*-(3-dimethylaminopropyl)-*N*′-ethylcarbodiimide hydrochloride (EDC), 3-morpholino-2-hydroxypropanesulfonic acid (MOPSO), phenolphthalein, potassium persulfate, sodium salt of *N*-hydroxysulfosuccinimide (sulfo-NHS), phosphate-buffered saline (PBS, pH 7.4), and thionine acetate (Th) were purchased from Sigma-Aldrich (St. Louis, MO, USA). Potassium permanganate, sodium hydroxide, hydrogen peroxide (30%), hydrochloric acid, and sulfuric acid were obtained from Lach-Ner (Neratovice, Czech Republic). All the chemicals used in the assay were of analytical grade. *N,N*-Diethyl-*p*-phenylenediamine sulfate salt (DPD; ≥98%) was purchased from Merck (Darmstadt, Germany). The DPD (12.53 mM) solution was prepared by dissolving a requisite quantity in deionized water. Ultrapure Q-water was from a Milli-Q Gradient A10 system (Millipore; Molsheim, France).

### Preparation of the ferrofluid

Water-based magnetic fluid stabilized with nitric acid was prepared using a modified Massart procedure^28^. FeCl_3_ ·6H_2_O (10.87 g) was dissolved in water (435 ml) at 80 °C with stirring and 1.5 M HCl (22 ml) containing FeCl_2_ ·4H_2_O (3.92 g) was added. 25% NH_4_OH (45 ml) was added rapidly and the black precipitate was formed, which was sedimented on a magnet and the supernatant was removed. 2 M Nitric acid (70 ml) was added at room temperature with stirring and the supernatant was decanted after the sedimentation for 10 min. The sediment was mixed with 0.33 M Fe(NO_3_)_2_ (75 ml) and the suspension was heated at 100 °C for 30 min. After cooling and sedimentation, the supernatant was removed and the iron oxide was washed with acetone several times. The concentration of nitric acid-stabilized water-based magnetic fluid was 50.3 mg/ml.

### Preparation of the P(GMA-EDMA) particles

Poly(glycidyl methacrylate-*co*-ethylene dimethacrylate) [P(GMA-EDMA)] particles were prepared by surfactant-free emulsion polymerization of glycidyl methacrylate (GMA) and an ethylene dimethacrylate (EDMA) crosslinker by modification of an earlier procedure^27^. Briefly, water (75 g), K_2_S_2_O_8_ initiator (0.1875 g), GMA (5.25 g) and EDMA (0.0158 g) were stirred (500 rpm) at 80 °C for 18 h, and the resulting particles were washed six times with water (50 ml each).

### Hydrolysis of the P(GMA-EDMA) particles and their oxidation

P(GMA-EDMA) particles (1 g) were hydrolyzed in 1 M H_2_SO_4_ (20 ml) at room temperature (RT) for 2 h. The hydrolyzed particles were added into aqueous KMnO_4_ (1.6 g, 80 ml) and the reaction proceeded at RT for 18 h. The particles were washed with 10% oxalic acid (20 ml) and five times with water to reach pH 7. The resulting poly(carboxymethyl methacrylate-*co*-ethylene dimethacrylate) [P(CMMA-EDMA)] particles were stirred in 1 M HCl (15 ml) at 20 °C for 7 h and washed five times with water.

### Attachment of thionine acetate to the P(CMMA-EDMA) particles

The P(CMMA-EDMA) particles (2 g) were dispersed in a mixture of 0.1 M MOPSO buffer (50 ml; pH 9), EDC·HCl (2.4 mg), and sulfo-NHS (1.45 mg) for 0.5 h at 0 °C. Th (3.62 mg) was added slowly, and the mixture (pH 9) was stirred at RT for an additional 48 h. The reaction was terminated by addition of 0.5 M NaOH to increase the pH to 11. The resulting P(CMMA-EDMA)-Th particles were washed with 0.01 M HCl (100 ml each) and then washed five times with 0.1 M PBS buffer (50 ml each; pH 7.4).

### Modification of the polymer particles with ferrofluid

Magnetic modification of both P(GMA-EDMA) and P(CMMA-EDMA)-Th was performed in the following way. A suspension of the starting particles (50 mg/ml; 2 ml) was centrifuged, the sediment was washed with methanol three times, the particles (2 ml) were suspended in methanol (10 ml), and nitric acid-stabilized ferrofluid (1 ml) was added; the suspension was mixed on a rotary mixer (Dynal, Norway; 16 rpm) for 4 h. The magnetically modified polymer particles were then repeatedly washed with methanol and water, resuspended in water (2 ml), and stored at 4 °C. Before the determination of the peroxidase-like activity, the suspension was diluted 10 times.

### Determination of the peroxidase-like activity

The peroxidase-like activity of the ferrofluid-modified P(CMMA-EDMA) [denoted as Mag-P(CMMA-EDMA)], ferrofluid-modified P(CMMA-EDMA)-Th [denoted as Mag-P(CMMA-EDMA)-Th] particles, and the neat P(CMMA-EDMA) particles was determined in a reaction mixture containing acetate buffer (pH 6; 3.3 ml), 12.53 mM DPD solution (400 µl) and 2% H_2_O_2_ (200 µl). The reaction was initiated by adding the diluted particle suspension (100 μl) into the mixture, which was followed by incubation at an appropriate temperature for 5 min with stirring (16 rpm). The magnetic particles were separated using a MPC-6 magnetic separator (Dynal, Norway), while nonmagnetic P(CMMA-EDMA) particles were separated by centrifugation (Hettich Universal 320, Germany) at 5,000 rpm for 5 min. The change in the absorbance of the supernatants was spectrophotometrically detected at 551 nm against the corresponding control containing the reagents, except for the polymer particles. To determine the optimal pH, acetate (pH 4–6), phosphate (pH 7–8), and carbonate (pH > 8) buffers were used, and the reaction was performed at 23 °C.

### Characterization

The morphology of the particles was investigated using a transmission electron microscope (TEM; JEOL 2000 FX) equipped with an energy dispersive X-ray spectrometer (EDAX; Mahwah, NJ, USA).

The size and distribution of dry particles were analyzed by scanning electron microscopy (SEM; Quanta 200 FEG). The number-average diameter (*D*_n_), weight-average diameter (*D*_w_), and uniformity characterized by dispersity (*Ð* = *D*_w_/*D*_n_) were calculated using ImageJ software by counting at least 500 individual particles in the SEM micrographs.

1 $${D}_{{\rm{n}}}={\sum }_{{{\rm{n}}}_{{\rm{i}}}}{D}_{{\rm{i}}}/{\sum }_{{{\rm{n}}}_{{\rm{i}}}}$$

2 $${D}_{{\rm{n}}}={\sum }_{{{\rm{n}}}_{{\rm{i}}}}{D}_{{\rm{i}}}^{4}/{\sum }_{{{\rm{n}}}_{{\rm{i}}}}{D}_{{\rm{i}}}^{3}$$

where n_i_ and *D*_i_ are the number and diameter of the i-th microsphere, respectively. The same microscope equipped with an energy dispersive X-ray spectrometer (Mahwah, NJ, USA) was used for determination of energy dispersive X-ray spectra (EDX).

The carboxyl group (*CG*) content in the P(CMMA-EDMA) particles was determined via a titration method^29^. The particles (60 mg) were completely hydrolyzed in 0.1 M NaOH (20 ml) at RT for 24 h with stirring, and the excess base was titrated with 0.1 M HCl using phenolphthalein as an indicator. The *CG* was calculated according to the following equation:

3 $$CG=[{c}_{{\rm{HCl}}}\cdot ({V}_{0}-{V}_{1})]/{\rm{m}}$$

where *c*_HCl_ is the concentration of HCl, *V*_0_ is the volume of 0.1 M NaOH, *V*_1_ is the volume of 0.1 M HCl needed for titration, and *m* is the weight of the P(CMMA-EDMA) particles.

Fourier transform infrared spectra (FTIR) were measured in attenuated total reflection (ATR) mode using a Thermo Nicolet NEXUS 870 FTIR spectrometer (Madison, WI, USA).

X-ray photoelectron spectra (XPS) were obtained using a K-Alpha^+^ XPS spectrometer (ThermoFisher Scientific, UK) operating at a base pressure of 1.0 × 10^−7^ Pa. The data acquisition and processing were performed using Thermo Avantage software. All particles were analyzed using microfocused and monochromated Al Kα X-ray radiation (400 μm spot size) with a pass energy of 200 eV for survey and 50 eV for high-energy resolution core level spectra. The X-ray angle of incidence was 30°, and the emission angle was along the surface normal. K-Alpha charge dual compensation system was employed during analysis by using electrons and low-energy argon ions to prevent localized charge build-up. The analyzer transmission function, Scofield sensitivity factors, and effective attenuation lengths (EALs) for the photoelectrons were applied for quantification. The EALs were calculated using the standard TPP-2 M formalism. The binding energy scale of the XPS spectrometer was calibrated by the well-known positions of the C 1s C–C, C–H, C–O and C(=O)–O peaks of poly(ethylene terephthalate) and Cu 2p, Ag 3d, and Au 4 f peaks of Cu, Ag and Au metals, respectively. The spectra were charge referenced to the C 1 s contribution at a binding energy of 285.0 eV attributed to the C–C and C–H moieties.

The magnetization was measured using an EV9 vibrating sample magnetometer (VSM; DSM Magnetics ADE Corporation; Lowell, MA, USA) at RT. The absorbance was recorded with a Cintra 20 UV/Vis spectrophotometer (GBC; Braeside, Australia) at 551 nm.

## Results and Discussion

### Preparation and modification of the poly(glycidyl methacrylate-co-ethylene dimethacrylate) particles

In our previous report, thionine-modified PGMA-based particles were found to be highly sensitive to the universal antibody labels in the sandwich-type electrochemical immunosensor, and they improved its electrochemical behavior^27^. In this work, novel magnetic P(GMA-EDMA) particles obtained by emulsifier-free emulsion polymerization and modification with a ferrofluid were investigated in terms of enhanced peroxidase-like activity. Crosslinking with EDMA was used to prevent aggregation of the particles in water and to improve their chemical stability in an acidic or basic environment, thus enabling thus prolonged hydrolysis and oxidation to increase the COOH content^30^. To maintain the spherical character and narrow size distribution of the P(GMA-EDMA) particles, the effect of a low crosslinker concentration from 0.3 to 0.7 wt.% of EDMA on the final particle size and morphology was investigated. Of note, in analogous polystyrene microspheres, only a limited amount of crosslinker (typically <1 wt.% of divinylbenzene) was allowed to be incorporated into the microspheres without losing their spherical shape and monodispersity^31^.

**Figure 1:**
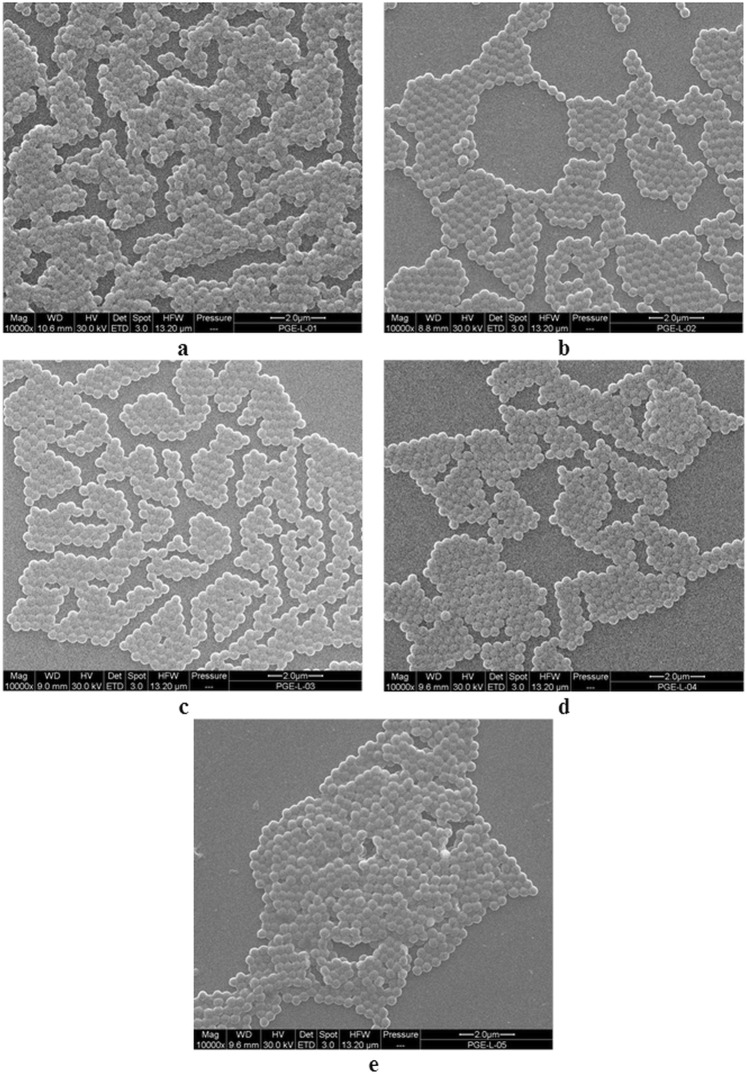
SEM micrographs of (**a**–**e**) P(GMA-EDMA)-1-5 particles.

According to SEM, the particles were spherical with a very narrow particle size distribution (*Ð* ~1.02; Table 1) and non-aggregated (Fig. 1a–e). The number-average diameter (*D*_n_) of the P(GMA-EDMA)-1–5 particles increased from 302 to 339 nm with increasing EDMA concentration (Table 1). This was in contrast to the decrease in poly(methyl methacrylate-*co*-ethylene dimethacrylate) particle size with increasing EDMA concentration at >10 wt.% of EDMA, which was explained by the enhanced polymerization rate and limited particle growth due to the reduced swellability of the crosslinked particles^32,33^. However, in this report, with increasing EDMA amounts, the monomer concentration increased, obviously leading to the formation of smaller number of oligomer radicals but with a longer critical length, which precipitated and produced larger mature particles. For the next experiments, namely, the introduction of carboxyl groups and the immobilization of thionine, only P(GMA-EDMA)-4 particles (*D*_n_ = 332 nm; Fig. 1d) prepared with 0.6 wt.% of EDMA were selected because they had the narrowest size distribution.

**Table 1:** Effect of EDMA concentration (*c*_EDMA_) on size of the P(GMA-EDMA) particles and their distribution.

Particles	*c*_EDMA_ (wt.%)	*D*_n_ (nm)	*Ð*
P(GMA-EDMA)-1	0.3	302 ± 26	1.022
P(GMA-EDMA)-2	0.4	315 ± 27	1.021
P(GMA-EDMA)-3	0.5	337 ± 28	1.021
P(GMA-EDMA)-4	0.6	332 ± 27	1.019
P(GMA-EDMA)-5	0.7	339 ± 29	1.022

*D*_n_ – number-average particle diameter (SEM), *Ð* – dispersity (SEM).

**Figure 2:**
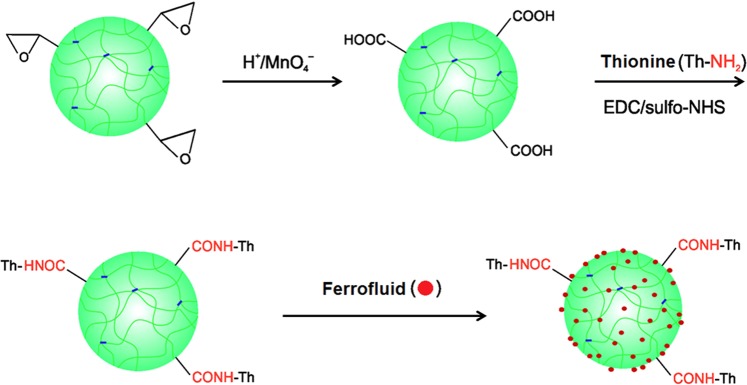
Scheme of hydrolysis and oxidation of the P(GMA-EDMA) particles, attachment of thionine acetate, and modification with nitric acid-stabilized ferrofluid.

The P(GMA-EDMA) particles were hydrolyzed with sulfuric acid and then oxidized using KMnO_4_ to introduce functional carboxyl groups (Fig. 2). Their content in the P(CMMA-EDMA) particles was calculated to be 0.66 mmol/g.

Because electron mediators can improve the electrical connection in biosensors^34^, the P(CMMA-EDMA) particles were also modified with thionine using EDC/sulfo-NHS chemistry. The attachment of thionine onto the particles was confirmed by a color change from white to violet-blue.

### Modification of the P(CMMA-EDMA) particles with ferrofluid

The existing literature describes various polymer-coated inorganic oxides that serve as artificial peroxidase mimetics^35–37^. However, a reverse system based on oxide-coated polymer particles has not yet been reported. For this reason, we attempted to develop this type of artificial peroxidase mimetic from P(CMMA-EDMA) polymer particles.

**Figure 3:**
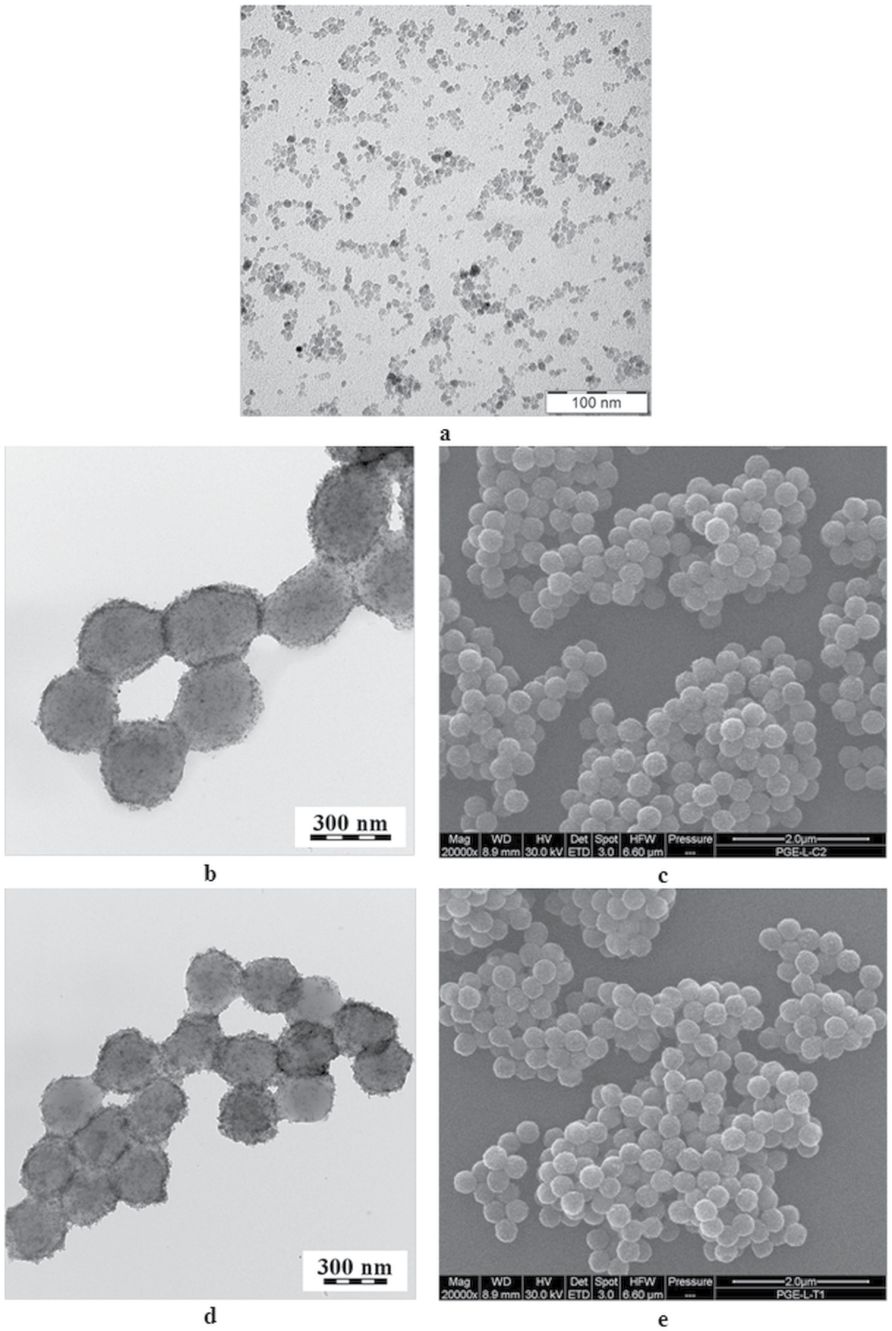
TEM (**a**,**b**,**d**) and SEM (**c**,**e**) micrographs of (**a**) ferrofluid, (**b**,**c**) Mag-P(CMMA-EDMA), and (**d**,**e**) Mag-P(CMMA-EDMA)-Th particles.

The P(CMMA-EDMA) and the P(CMMA-EDMA)-Th particles were magnetically modified with the nitric acid-stabilized ferrofluid in methanol (Fig. 2). Ferrofluid consisted of iron oxide nanoparticles with an average diameter *D*_n_ = 5.5 nm (Fig. 3a, Table 2). The dispersity (*Đ*) was rather broad, the particle size ranged from 2 to 11 nm. According to EDX spectroscopy, the particles contained 25.9% of Fe; the ferrofluid had high saturation magnetization (*M*_s_ = 43 Am^2^/kg).

**Table 2:** Characterization of the magnetic particles.

Particles	*D*_n_ (nm)	*Ð*	Fe (wt.%)^a^	*H*_c_ (A/m)	*M*_rs_ (A m^2^/kg)	*M*_s_ (A m^2^/kg)
Ferrofluid	5.5 ± 1.5	1.244	25.9	−^b^	−^b^	43.1
Mag-P(CMMA-EDMA)	330 ± 20	1.010	15.3	4.38	2.09·10^−3^	11.1
Mag-P(CMMA-EDMA)-Th	328 ± 23	1.011	14.3	9.79	3.36·10^−3^	10.0

^a^Surface composition (ferrofluid by EDX and Mag-P(CMMA-EDMA) by XPS), *D*_n_ - number-average particle diameter, *Ð* – dispersity, *H*_c_ - coercive force, *M*_rs_ - saturation remanent magnetization, *M*_s_ - saturation magnetization (VSM). ^b^Negligible (on the noise level).

**Figure 4:**
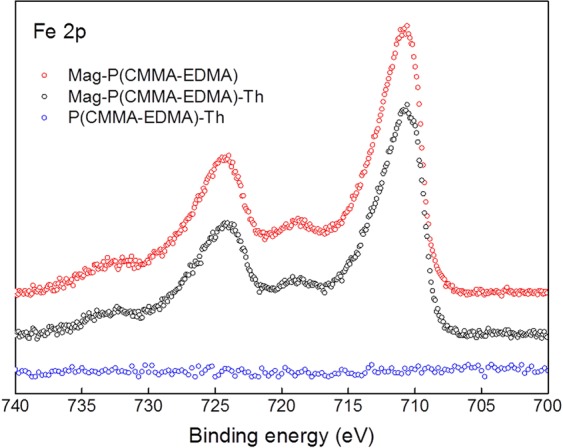
High resolution core-level Fe 2p XPS spectra of the P(CMMA-EDMA)-Th (blue), Mag-P(CMMA-EDMA)-Th (black) and Mag-P(CMMA-EDMA) particles (red).

After introduction of the ferrofluid on the P(CMMA-EDMA) and P(CMMA-EDMA)-Th particles, the surface changed from smooth to the rough one, as documented on both TEM and SEM images (Figs 1 and 3). TEM micrographs detected complete decoration of the methacrylate-based particles with the ferrofluid (Fig. 3). The Mag-P(CMMA-EDMA)-Th particle size (*D*_n_ ∼330 nm) barely changed from that of the starting P(CMMA-EDMA) particles (Table 2; Fig. 3). The particles seem to be smaller on TEM images than on SEM micrographs due to the particle shrinking, when the sample is dried on TEM. A high amount of iron oxide nanoparticles was found on the surface of the Mag-P(CMMA-EDMA) and Mag-P(CMMA-EDMA)-Th particles according to TEM (Fig. 3). XPS spectroscopy and VSM was then used to quantify the amount of Fe on the particles (Table 2). Modification by ferrofluid was confirmed by the Fe 2p signals in the XPS spectra of the magnetic particles (Fig. 4). The position of Fe 2p_3/2_ peak in high resolution XPS spectra (binding energy 710.9 eV for both Mag-P(CMMA-EDMA) and Mag-P(CMMA-EDMA)-Th) suggested that the iron oxide was maghemite (Fe 2p_3/2_ peak at 710.8 eV). The higher amount of Fe on the Mag-P(CMMA-EDMA) particles according to XPS compared with that obtained by TEM energy-dispersive X-ray spectroscopy (data not shown) also indicated that modification preferably occurred on the particle surface.

**Figure 5:**
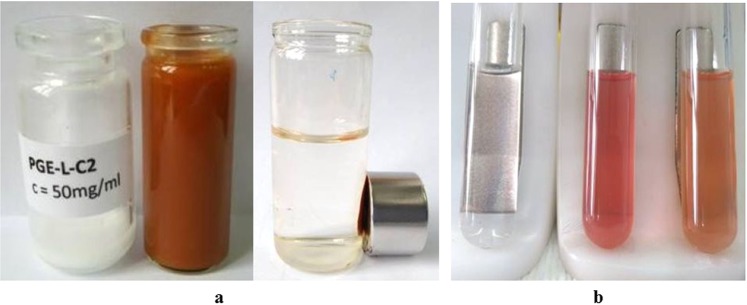
Light photographs documenting (**a**) the magnetic separation of Mag-P(CMMA-EDMA) particles (from left to right: P(CMMA-EDMA) and Mag-P(CMMA-EDMA) particles twice) and (**b**) the peroxidase-like activity of the blank, Mag-P(CMMA-EDMA), and Mag-P(CMMA-EDMA)-Th particles (from left to right).

The magnetic properties of the Mag-P(CMMA-EDMA) and Mag-P(CMMA-EDMA)-Th particles were measured using a VSM (Table 2). The coercivity and magnetic remanence were negligible on the limit of sensitivity and accuracy of the measurement and were 1–2 orders lower than the values reported for 10-nm maghemite particles produced by a plasma technique^38^ or coprecipitation in air^39^. This can be explained by the rather thin layer of magnetic iron oxide nanoparticles on the particle surface. Both values suggest that the particles were noninteracting and superparamagnetic. The *M*_s_ values of Mag-P(CMMA-EDMA) and Mag-P(CMMA-EDMA)-Th particles were lower than that of the neat ferrofluid: 11.1 and 10.0 Am^2^/kg, respectively (Table 2). Supposing that the ferrofluid is composed of maghemite (*M*_s_ = 80 A m^2^/kg)^40^, this indicates that 13.9 and 12.5 wt.% of γ-Fe_2_O_3_ was present on the particles. This estimated iron oxide content is lower than that determined via the surface XPS analysis (15.3 and 14.3 wt.% of Fe represent 21.9 and 20.4 wt.% of Fe_2_O_3_). However, it was quite sufficient for magnetic separation (Fig. 5a). Using a NdFeB permanent magnet (cylinder 20 × 10 mm, remanence 1.3 T), it was possible to collect magnetically modified particles in standard test tubes (diameter of 10 mm) in less than 30 s.

### Peroxidase-like activity of the Mag-P(CMMA-EDMA) and Mag-P(CMMA-EDMA)-Th particles

**Figure 6:**
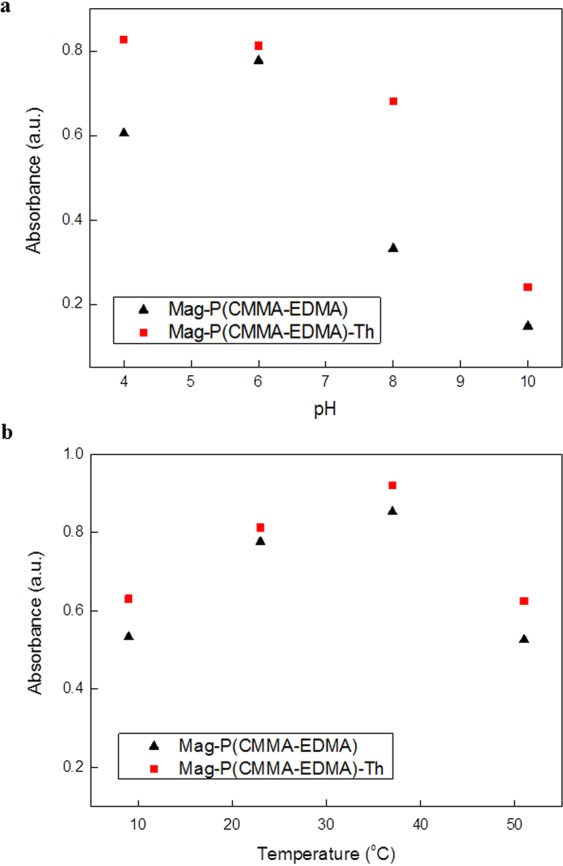
(**a**) pH and (**b**) temperature dependence of the peroxidase-like activity of Mag-P(CMMA-EDMA) and Mag-P(CMMA-EDMA)-Th particles. The absorbance was recorded at 551 nm. Polynomial fitting models were obtained using an Origin 9.1 software and the following equations were calculated: (**a**) Mag-P(CMMA-EDMA): y = 0.01829x^3^ − 0.40638x^2^ + 2.75958x − 5.0102; Mag-P(CMMA-EDMA)-Th: y = −0.00404x^3^ + 0.05825x^2^ − 0.28283x + 1.285 and (**b**) Mag-P(CMMA-EDMA): y = −0.0006x^2^ + 0.0372x + 0.3289; Mag-P(CMMA-EDMA)-Th: y = − 0.0007x^2^ + 0.0441x + 0.00822.

It has been recently shown that specific magnetic nanoparticles and their polymer-coated composites provide peroxidase-like activity, which enables oxidation of various substrates in the presence of hydrogen peroxide^1,41^. Also the carboxyl groups can enhance the enzymatic activity, while the amino groups decrease it^42^. Moreover, electron-transfer mediators, such as thionine, can intensify the electron transfer^34^. Therefore, we constructed two types of artificial peroxidase mimetics, Mag-P(CMMA-EDMA) and Mag-P(CMMA-EDMA)-Th particles, to study their peroxidase-like activity. The activity was determined via a photometric method in which the well-known chromogenic substrate (*N,N*-diethyl-1,4-phenylenediamine; DPD) was oxidized by a peroxidase-like catalyzed reaction. The formation of the purple-colored reaction product was measured spectrophotometrically at 551 nm (Fig. 5b). The neat P(CMMA-EDMA) particles did not show significant peroxidase-like activity, confirming the importance of bound magnetic iron oxide for achieving the abovementioned enzyme-like activity. The pH dependence of the enzymatic activity revealed that Mag-P(CMMA-EDMA) has a maximum peroxidase-like activity at pH ~6, whereas the Mag-P(CMMA-EDMA)-Th particles are enzymatically active over a wider pH range (Fig. 6a). Both Mag-P(CMMA-EDMA) and Mag-P(CMMA-EDMA)-Th exhibited similar high peroxidase-like activities at ∼37 °C (Fig. 6b). The improved enzymatic activity was probably caused by the fact that the immobilized thionine supported the electron transfer between the ferrofluid attached on the particles and the substrate. The optimal temperature and pH values of both types of magnetic particles correspond to values found by other researchers^43^. The specific peroxidase-like activity of magnetically modified particles under optimal reaction conditions (pH 6, 37 °C) was 6.50 µkat/ml for sedimented Mag-P(CMMA-EDMA) particles and 7.02 µkat/ml for sedimented Mag-P(CMMA-EDMA)-Th particles.

In conclusions, Mag-P(CMMA-EDMA)-Th particles were synthesized via emulsifier-free emulsion polymerization, immobilization of thionine, and modification with a ferrofluid. The effect of EDMA concentration on the starting P(CMMA-EDMA) particle size and size distribution was studied; the particle size changed from 300 to 340 nm with increasing EDMA concentration, and the particles were monodisperse. The incorporation of a low EDMA concentration allowed an increase in the reaction time during hydrolysis and the oxidation of the particles in the presence of sulfuric acid without damaging the particle. This resulted in a relatively high content of carboxyl groups in the P(CMMA-EDMA) particles. The particles were additionally modified with thionine to endow them with a higher electron transfer activity and improved peroxidase-like activity. Both types of particles, namely, P(CMMA-EDMA) and P(CMMA-EDMA)-Th, were successfully modified with ferrofluid. The highest saturation magnetization of the magnetic particles was 11 Am^2^/kg. Both types of artificial peroxidase mimetics based on the ferrofluid-coated P(CMMA-EDMA) particles exhibited enzyme-like activity, which had a maximum at 37 °C and pH ~6. Modification of the particles with thionine improved their pH stability, resulting in an expanded maximum enzymatic activity over a wider pH range. Our results suggest that these ferrofluid-coated polymer particles could be promising in the development of a highly sensitive sensor in an artificial enzyme-based immunoassay.

## References

[1] Wei H, Wang E (2008). Fe_3_O_4_ magnetic nanoparticles as peroxidase mimetics and their applications in H_2_O_2_ and glucose detection. Anal. Chem..

[2] Mu J, Li J, Zhao X, Yang EC, Zhao X-J (2016). Cobalt-doped graphitic carbon nitride with enhanced peroxidase-like activity for wastewater treatment. RSC Adv..

[3] Li M (2017). Dichlorofluorescein as a peroxidase mimic and its application to glucose detection. New J. Chem..

[4] Wang S (2017). Copper-based metal–organic framework nanoparticles with peroxidase-like activity for sensitive colorimetric detection of *Staphylococcus aureus*. ACS Appl. Mater. Interfaces.

[5] Jiang Y, Song N, Wang C, Pinna N, Lu X (2017). A facile synthesis of Fe_3_O_4_/nitrogen-doped carbon hybrid nanofibers as a robust peroxidase-like catalyst for the sensitive colorimetric detection of ascorbic acid. J. Mater. Chem. B.

[6] Wang N, Sun J, Chen L, Fan H, Ai S (2015). A Cu_2_(OH)_3_Cl-CeO_2_ nanocomposite with peroxidase-like activity, and its application to the determination of hydrogen peroxide, glucose and cholesterol. Microchim. Acta.

[7] Ci YX, Chen L, Wei S (1989). Fluorescence reaction of the system mimetic peroxidase [Mn-T(4-TAP)P] - homovanillic acid-hydrogen peroxide. Spectrofluorimetric determination of H_2_O_2_. Fresenius J. Anal. Chem..

[8] Sun L, Ding Y, Jiang Y, Liu Q (2017). Montmorillonite-loaded ceria nanocomposites with superior peroxidase-like activity for rapid colorimetric detection of H_2_O_2_. Sens Actuators B Chem..

[9] Fu S (2017). Structural effect of Fe_3_O_4_ nanoparticles on peroxidase-like activity for cancer therapy. Colloids Surf. B Biointerfaces.

[10] Li K, Zhao Y, Janik MJ, Song C, Guo X (2017). Facile preparation of magnetic mesoporous Fe_3_O_4_/C/Cu composites as high performance Fenton-like catalysts. Appl. Surf. Sci..

[11] Gao L (2007). Intrinsic peroxidase-like activity of ferromagnetic nanoparticles. Nat. Nanotechnol..

[12] Lin Y, Ren J, Qu X (2014). Catalytically active nanomaterials: a promising candidate for artificial enzymes. Acc. Chem. Res..

[13] Golchin J (2017). Nanozyme applications in biology and medicine: an overview. Artif. Cells Nanomed. Biotechnol..

[14] An Q (2013). Peroxidase-like activity of Fe_3_O_4_@carbon nanoparticles enhnaces ascorbic acid-induced oxidative stress and selective damage to PC-3 prostate cancer cells. ACS Appl. Mater. Interfaces.

[15] Chaudhari KN, Chaudhari NK, Yu JS (2012). Peroxidase mimic activity of hematite iron oxides (α-Fe_2_O_3_) with different nanostructures. Catal. Sci. Technol..

[16] Palmqvist, N. G. M., Seisenbaeva, G. A., Svedlindh, P. & Kessler, V. G. Maghemite nanoparticles acts as nanozymes, improving growth and abiotic stress tolerance in Brassica nanpus. *Nanoscale Res. Lett*. **12**, 10.1186/s11671-017-2404-2 (2017). 10.1186/s11671-017-2404-2 PMC5736512 29260423

[17] Melnikova L, Pospiskova K, Mitroova Z, Kopcansky P, Safarik I (2014). Peroxidase-like activity of magnetoferritin. Microchim. Acta.

[18] Horák D, Babič M, Macková H, Beneš MJ (2007). Preparation and properties of magnetic nano- and microsized particles for biological and environmental separations. J. Sep. Sci..

[19] Zhang K (2017). Multi-layer dextran-decorated poly(glycidyl methacrylate)-*co*-divinyl benzene copolymer matrices enabling efficient protein chromatographic separation. React. Funct. Polym..

[20] Yu B, Tian C, Cong H, Xu T (2016). Synthesis of monodisperse poly(styrene-co-divinylbenzene) microspheres with binary porous structures and application in high-performance liquid chromatography. J. Mater. Sci..

[21] Tasfiyati AN, Iftitah ED, Sakti SP, Sabarudin A (2016). Evaluation of glycidyl methacrylate-based monolith functionalized with weak anion exchange moiety inside 0.5 mm i.d. column for liquid chromatographic separation ofDNA. Analytical. Chem. Res..

[22] Nastasović AB (2016). Mechanism of Cu(II), Cd(II) and Pb(II) ions sorption from aqueous solutions by macroporous poly(glycidyl methacrylate-*co*-ethylene glycol dimethacrylate). Appl. Surf. Sci..

[23] Ying L-L (2017). Poly(glycidyl methacrylate) nanoparticle-coated capillary with oriented antibody immobilization for immunoaffinity in-tube solid phase microextraction: Preparation and characterization. J. Chromatogr. A.

[24] Zasońska, B. A. *et al*. Monodisperse magnetic poly(glycidyl methacrylate) microspheres for isolation and determination of blood serum immunoglobulins with affinity to short form of unconventional Myo1C. *Microchim. Acta* **185**, 10.1007/s00604-018-2807-5 (2018). 10.1007/s00604-018-2807-5 29687337

[25] Yang HH (2004). Magnetite-containing spherical silica nanoparticles for biocatalysis and bioseparations. Anal. Chem..

[26] Soh N (2004). Chemiluminiscence sequential injection immunoassay for vitellogenin using magnetic microbeads. Talanta.

[27] Zasońska BA (2015). Thionine-modified poly(glycidyl methacrylate) nanospheres as labels of antibodies for biosensing applications. ACS Appl. Mater. Interfaces.

[28] Massart R (1981). Preparation of aqueous magnetic liquids in alkaline and acidic media. IEEE Trans. Magn..

[29] Heydari D, Sheibari H (2016). Facile polymerization of β-cyclodextrin functionalized graphene or graphene oxide nanosheets using citric acid crosslinker by *in situ* melt polycondensation for enhanced electrochemical performance. RSC Adv..

[30] Zhang J, Wang LL, Ma JQ, Wang YL (2014). Preparation of ofloxacin poly(glycidyl methacrylate-*co*-ethylenedimethacrylate) (P_GMA/EDMA_) molecularly imprinted microspheres and their application to the analysis of quinolones in milk. Food Anal. Methods.

[31] Song JS, Winnik MA (2005). Cross-linked, monodisperse, micron-sized polystyrene particles by two-stage dispersion polymerization. Macromolecules.

[32] Tanrisever T, Okay O, Sönmezoglu IC (1996). Kinetics of emulsifier-free emulsion polymerization of methyl methacrylate. J. Appl. Polym. Sci..

[33] Sajjadi S (2015). Extending the limits of emulsifier-free emulsion polymerization to achieve small uniform particles. RSC Adv..

[34] Ruan C, Yang F, Lei C, Deng J (1998). Thionine covalently tethered to multilayer horseradish peroxidase in a self-assembled monolayer as an electron-transfer mediator. Anal. Chem..

[35] Asati A, Santra S, Kaittanis C, Nath S, Perez JM (2009). Oxidase-like activity of polymer-coated cerium oxide nanoparticles. Angew. Chem. Int. Ed..

[36] Yu F, Huang Y, Cole AJ, Yang VC (2009). The artificial activity of magnetic iron oxide nanoparticles and its application to glucose detection. Biomaterials.

[37] He W, Wamer W, Xia Q, Yin JJ, Fu PP (2014). Enzyme-like activity of nanomaterials. Environ. Carcinog, Ecotoxical Rev.-Pt. C J. Env. Sci. Health.

[38] Sarma, L. *et al*. Size-controlled synthesis of superparamagnetic iron-oxide and iron-oxide/iron/carbon nanotube nanocomposites by supersonic plasma expansion technique. *J. Phys. D Appl. Phys*. **51**, 10.1088/1361-6463/aaba93 (2018).

[39] Rebolledo UA, Nandini S, Sanchez OE, Sarma SSS (2018). Combined effects of temperature and salinity on the demographic response of Proales similis (Beauchamp, 1907) and Brachionus plicatilis (Muller, 1786) (Rotifera) to mercury. Chemosphere.

[40] Dunlop, D. & Ozdemir, O. *Rock Magnetism: Fundamentals and Frontiers* (Cambirdge Univ. Press 1997).

[41] Wei H, Wang E (2013). Nanomaterials with enzyme-like characteristics (nanozymes): Next-generation artificial enzymes. Chem. Soc. Rev..

[42] Liu L (2017). Effect of carboxyl and amino groups in fluorescein molecules on their peroxidase-like aktivity, Mol. Catal..

[43] Gao L, Fan K, Yan X (2017). Iron oxide nanozyme: A multifunctional enzyme mimetic for biomedical applications. Theranostics.

